# *In Vitro* Metabolic and Mitogenic Signaling of Insulin Glargine and Its Metabolites

**DOI:** 10.1371/journal.pone.0009540

**Published:** 2010-03-04

**Authors:** Mark R. Sommerfeld, Günter Müller, Georg Tschank, Gerhard Seipke, Paul Habermann, Roland Kurrle, Norbert Tennagels

**Affiliations:** 1 Research and Development TD Metabolism, Sanofi-Aventis Deutschland GmbH, Frankfurt am Main, Germany; 2 Process Development Biotechnology, Sanofi-Aventis Deutschland GmbH, Frankfurt am Main, Germany; University of Bremen, Germany

## Abstract

**Background:**

Insulin glargine (Lantus®) is a long-acting basal insulin analog that demonstrates effective day-long glycemic control and a lower incidence of hypoglycemia than NPH insulin. After subcutaneous injection insulin glargine is partly converted into the two main metabolites M1 ([Gly^A21^]insulin) and M2 ([Gly^A21^,des-Thr^B30^]insulin). The aim of this study was to characterize the glargine metabolites *in vitro* with regard to their insulin receptor (IR) and IGF-1 receptor (IGF1R) binding and signaling properties as well as their metabolic and mitogenic activities.

**Methods:**

The affinity of human insulin, insulin glargine and its metabolites to the IR isoforms A and B or IGF1R was analyzed in a competitive binding assay using SPA technology. Receptor autophosphorylation activities were studied via In-Cell Western in CHO and MEF cells overexpressing human IR-A and IR-B or IGF1R, respectively. The metabolic response of the insulins was studied as stimulation of lipid synthesis using primary rat adipocytes. Thymidine incorporation in Saos-2 cells was used to characterize the mitogenic activity.

**Conclusions:**

The binding of insulin glargine and its metabolites M1 and M2 to the IR were similar and correlated well with their corresponding autophosphorylation and metabolic activities *in vitro*. No differences were found towards the two IR isoforms A or B. Insulin glargine showed a higher affinity for IGF1R than insulin, resulting in a lower EC_50_ value for autophosphorylation of the receptor and a more potent stimulation of thymidine incorporation in Saos-2 cells. In contrast, the metabolites M1 and M2 were significantly less active in binding to and activation of the IGF1R and their mitogenicity in Saos-2 cells was equal to human insulin. These findings strongly support the idea that insulin glargine metabolites contribute with the same potency as insulin glargine to blood glucose control but lead to significantly reduced growth-promoting activity.

## Introduction

Insulin glargine (Lantus®, [Gly^A21^,Arg^B31^,Arg^B32^]insulin) is a long-acting human insulin analog with an almost peakless activity profile very closely mimicking the natural physiological profile of basal endogenous insulin secretion [1], [1]–[5]. Insulin glargine differs from human insulin by substitution of asparagine by glycine in position 21 of the A chain and by carboxy-terminal extension of the B-chain by two arginine residues. These alterations cause a shift in the isoelectric point from pH 5.4 to 6.7. Following subcutaneous administration as a clear solution of pH 4, insulin glargine precipitates at the injection site because of its low solubility at physiological pH. The prolonged blood glucose lowering activity of insulin glargine may be a consequence of the subsequent slow dissolution of the microprecipitate on the basis of a low dissociation rate [6]. However, the extended action profile of insulin glargine may be dependent on more than just a shift in its isoelectric point [7], [8].

**Figure 1:**
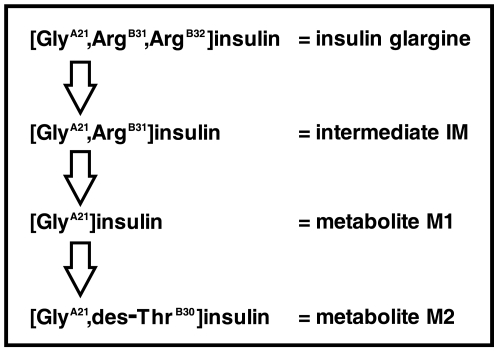
Insulin glargine metabolites. The long-acting insulin glargine (Lantus®, [Gly^A21^,Arg^B31^,Arg^B32^]insulin) is metabolized *in vivo* in subcutaneous tissue and in bloodstream of healthy humans by sequential cleavage of the C-terminus of the B chain. Two primary degradation products M1 and M2 have been reported, which are both structural similar to human insulin [10]. Besides M1 and M2 an intermediate IM was also identified but only in minor quantities.

Insulin glargine may be released from the microprecipitate by proteolytic degradation leading to soluble yet fully metabolically active metabolites. *In vivo* metabolism studies in rats and dogs have demonstrated significant plasma levels of two main metabolites of insulin glargine, M1 ([Gly^A21^]insulin) and M2 ([Gly^A21^,des-Thr^B30^]insulin), formed by the sequential removal of the two arginines from the carboxy-terminus of the B-chain and additional deamination of the threonine in position B30. The intermediate IM ([Gly^A21^,Arg^B31^]insulin) can be detected as minor species only (Fig. 1) [9]. This pattern of insulin glargine metabolism was also observed following its subcutaneous administration in healthy humans [10]. Interestingly, degradation to both M1 and M2 occurred at the site of injection and continued within the circulatory system, although with considerable variation in efficacy and time course between the individuals tested. Analysis of tissue samples from the site of injection revealed an average ratio of 50∶50 for parent compound and M1/M2 [10]. *In vitro* incubation of insulin glargine in 69 sera for 30 minutes at 37°C resulted in metabolizing of insulin glargine into M1 from 46% to 98% [11]. The processing enzymes have not been identified so far, but metallocarboxypeptidases such as carboxypeptidases E, H, N and U represent candidates for the rapid and efficient metabolism of insulin glargine [11]. These enzymes are found in plasma and are likely to occur also in subcutaneous tissues [11].

The efficient generation of IM, M1, and M2 at the subcutaneous injection site and in plasma carries the notion that the proteolytic degradation products of insulin glargine contribute to the long-lasting systemic metabolic activity at least in part. In addition, it may shed new light on the current intense and controversial debate concerning the growth-promoting activity of human insulin and insulin analogs as determined *in vitro* [12]–[15] and its *in vivo* relevance for insulin therapy [16]–[18]. The growth-promoting effects of human insulin or insulin analogs are usually studied in cell lines under *in vitro* experimental conditions in the absence of IGF's and other physiological serum growth factors. However, several such studies have yielded conflicting results that may be due to experiment-to-experiment variability in specific cell lines and/or to the precise experimental conditions used [18], [19].

In addition, modification of human insulin by amino acid substitutions is known to change the signaling properties of the hormone. One prominent example is the aspartate modification in the B-chain of insulin, Asp^B10^, resulting in a higher affinity towards both IR and IGF1R and a prolonged occupancy time for the IR [12]. The fact that [Asp^B10^]insulin has induced a higher incidence of breast cancer in a 1-year toxicity study in Sprague-Dawley rats [20] and a higher proliferation rate of the osteosarcoma cell line Saos-2 [12] has led to the contention that insulins with a profile *in vitro* similar to [Asp^B10^]insulin might also have a potential tumor-promoting activity.

The human insulin receptor is expressed as two isoforms as a result of alternative splicing. The two mature proteins IR-A (short form) and IR-B (long form) differ by the presence or absence of 12 amino acids encoded by exon 11 at the carboxy-terminus of the extracellular α-subunit, downstream of the carboxy-terminal sequence that is essential for ligand binding [21], [22]. IR-A appears to be expressed predominantly in fetal tissues and cancer, whereas IR-B is expressed in normal adult tissues [23]. Importantly, there is considerable evidence that IR-A, which has the peculiar characteristic to bind not only insulin but also IGF-2, may play a critical role in the development of breast cancer [24], thyroid cancer [25] and leiomyosarcoma [26]. In addition, a role of the isoform IR-A has been discussed for hybrid receptors, as increased expression of IR-A/IGF1R hybrids has been found in tumors [27].

Therefore it is important to determine the metabolic and mitogenic as well as receptor binding activities of insulin glargine metabolites, which so far have only been reported in part for M1 [12]. In the present study we provide IR- and IGF1R-mediated metabolic and mitogenic signaling profiles for insulin glargine, IM, M1, and M2, including the differentiation between IR-A and IR-B.

## Materials and Methods

### Materials

Human insulin, insulin glargine, insulin glargine intermediate IM ([Gly^A21^,Arg^B31^]insulin), M1 ([Gly^A21^]insulin), M2 ([Gly^A21^,des-Thr^B30^]insulin), and [Asp^B10^]insulin were produced by recombinant DNA techniques or enzymatic semisynthesis and purified to homogeneity and were made available by Process Development Biotechnology (Sanofi-Aventis, Frankfurt, Germany). Human A14[^125^I]-insulin was prepared by the radiosynthesis group of Sanofi-Aventis (Frankfurt, Germany) [28]. [2-^14^C]-thymidine was obtained from Perkin Elmer (Boston, MA, USA). IGF-1 and IGF-2 were obtained from R&D Systems (Wiesbaden-Nordenstadt, Germany) and from Cell Sciences (Canton, MA, USA). Complete Protease Inhibitor was from Roche (Penzberg, Germany). SPA PVT PEI treated WGA Beads were purchased from GE Healthcare (Amersham, UK). Cell culture reagents and antibodies were obtained from the suppliers as indicated in the methods section. All other chemicals were of reagent grade.

### Receptor Binding Assays

The binding of the different insulins to the human IR-A and IR-B was analyzed in a competitive binding assay using the scintillation proximity assay as previously described [29], [30]. Plasma membranes were enriched from CHO cells overexpressing either IR-A or IR-B (∼1.3×10^5^ IR-A per cell and ∼2.2×10^5^ IR-B per cell as determined by FACS analysis, unpublished data) by a series of differential centrifugations including a single flotation through a one-step sucrose gradient. Briefly, cells were grown to confluence and gently detached, transferred to a centrifugation tube followed by centrifugation for 10 minutes at 600×g at 4°C. The pellet was resuspended in ice-cold 2.25 STM buffer (2.25 mol/L sucrose, 5 mmol/L Tris-HCl pH 7.4, 5 mmol/L MgCl_2_, 1x Complete Protease Inhibitor) and disrupted using a hand-held Dounce homogenizer followed by sonication. This homogenate was transferred to a centrifugation tube, overlaid with 0.8 STM buffer (0.8 mol/L sucrose, 5 mmol/L Tris-HCl pH 7.4, 5 mmol/L MgCl_2_, 1x Complete Protease Inhibitor) and centrifuged for 90 minutes at 100 000×g at 4°C. The emerging pellicle at the interface was collected, transferred to a new tube and washed two times with PBS by centrifugation for 10 minutes at 1500×g. The final pellet was resuspended in a small volume of dilution buffer (50 mmol/L Tris-HCl pH 7.4, 5 mmol/L MgCl_2_, 1x Complete Protease Inhibitor) and homogenized with a Dounce homogenizer. These plasma membrane preparations were stored until usage at –80°C.

Binding experiments were conducted in 96-well microplates. Per well 2 µg of membranes were incubated with 0.25 mg wheat germ agglutinin polyvinyltoluene polyethylenimine SPA beads, 100 pmol/L A14[^125^I]-insulin and various concentrations of unlabeled insulins in a binding buffer containing 0.05 mol/L Tris-HCl pH 7.8, 0.15 mol/L NaCl, 0.1% BSA, Complete Protease Inhibitor for 12 hours at room temperature (23°C). The radioactivity was measured at equilibrium in a microplate scintillation counter (Wallac Microbeta, Freiburg, Germany).

### Receptor Autophosphorylation Assays

CHO cells expressing either the IR-A or the IR-B isoform were used for IR autophosphorylation assays using In-Cell Western [31]. For the analysis of IGF1R autophosphorylation the receptor was overexpressed in a mouse embryo fibroblast 3T3 Tet off cell line (BD Bioscience, Heidelberg, Germany) that was stably transfected with IGF1R tetracycline-regulatable expression plasmid resulting in the expression of ∼2.6×10^5^ IGF1R per cell (as determined by FACS analysis, unpublished data). In order to determine the receptor tyrosine phosphorylation level, cells were seeded into 96-well plates and grown for 48 h. Cells were serum starved with serum-free medium αMEM (PAN Biotech GmbH, Aidenbach, Germany) for 3–4 h. The cells were subsequently treated with increasing concentrations of either human insulin, IGF-1, IGF-2 or the indicated insulin analog for 15 min at 37°C. After incubation the medium was discarded and the cells were fixed in 3.75% freshly prepared para-formaldehyde for 20 min. Cells were permeabilized with 0.1% Triton-X-100 in PBS for 20 min. Blocking was performed with Odyssey blocking buffer (LI-COR, Bad Homburg, Germany) overnight at 4°C. Anti-pTyr 4G10 (Millipore, Schwalbach, Germany) was incubated for 2 h at room temperature. After incubation of the primary antibody, cells were washed with PBS+0.1% Tween20. The secondary anti-mouse-IgG-800-CW antibody (Rockland, Gilbertsville, PA, USA) was incubated for 1 h. Results were normalized by the quantification of DNA with TO-PRO3 dye (Invitrogen, Karlsruhe, Germany). Data were obtained as relative units (RU) and are presented as fold over basal.

### Metabolic Activity Assay

The metabolic activity of the different insulins was compared using insulin-stimulated lipid synthesis in primary rat adipocytes. Primary rat adipocytes were prepared according to published procedures [32], [33]. Briefly, rats (Sprague-Dawley, male, 140–160 g, fed ad libitum) were provided from Charles-River (Sulzfeld, Germany) and killed by cervical dislocation in accordance with the German animal protection law. The animals were used for the preparation of primary adipocytes, exclusively, and were not subjected to any pretreatment, such as drug administration or starvation. Epididymal fat pads were rapidly removed, stripped of blood vessels and placed in PBS. Subsequently, the pads were washed with KRBB bubbled with 5% O_2_/95% CO_2_. Each pad was then cut into two or three pieces. Two pieces of each were incubated with 1.5 mL of digestion buffer (10 mg collagenase [Worthington Inc., CLS, type I, 190 units/mg] in 10 ml of KRBB containing 9 mg glucose) for 15–30 min at 37°C in a shaking water bath (240 cycles/min). Released adipocytes were separated from residual undigested tissue by passage through a nylon web (mesh size 150 µm) and washed by flotation in a plastic tube two or three times with KRBB and adjusted to the appropriate titer of 3.5×10^5^ cells/mL. For distribution of the adipocytes, 200 µL of the suspension was removed under continuous gentle stirring with a plastic agitator.

Lipid synthesis was measured as the incorporation of [3-^3^H]glucose into toluene-extractable lipids, at a final cell titer of 0.7×10^4^ cells/mL in KRBB (1.2 mM KH_2_PO_4_, 1.2 mM MgSO_4_, 4.8 mM KCl, 25 mM NaHCO_3_, 120 mM NaCl, 2.4 mM CaCl_2_, containing 1% BSA [defatted, fraction V, Sigma, Deisenhofen, Germany] and 37 µM glucose and adjusted to pH 7.4) according to published procedures (Müller *et al.*, 2008) with the following modifications: 680 µL of KRBB were filled into 10-mL scintillation vials and supplemented with 100 µL of D-[3-^3^H]glucose (2 µCi/mL KRBB, 1 mM, Amersham-Buchler, Braunschweig, Germany) and 20 µL of insulin solution in KRBB. Lipid synthesis was started by the addition of 200 µL of adipocyte suspension in KRBB. After incubation for 90 min at 37°C under an atmosphere of 5% O_2_/95% CO_2_ and gentle shaking, 10 mL of toluene-based scintillation cocktail (Quickszint 501, Zinsser, Germany) was added. The samples were vigorously mixed and after phase separation (2 to 4 h) measured for radioactivity by liquid scintillation counting. Determination of the radioactivity contained in the toluene phase and corrected for non-specific spill-over of the radioactivity of unincorporated radiolabelled glucose contained in the water phase predominantly reflects the *de novo* lipid synthesis.

### Mitogenic Potency

The human osteosarcoma cell line Saos-2 was obtained in frozen aliquots from the European Collection of Cell Cultures (ECACC, Salesbury, UK). Cells were grown in McCoy's 5a medium (Gibco, Grand Island, NY, USA) supplemented with 10% fetal calf serum (PAN Biotech GmbH, Aidenbach, Germany) and 2 mM (final) L-glutamine (Sigma Aldrich, Irvine, UK). Subconfluent cultures (9-15×10^6^ cells per 225 cm^2^ flask) were used to determine the mitogenic activity of the test compounds.

For measuring thymidine incorporation 40,000 cells were seeded per well of a 96-well Cytostar-T scintillation microplate (GE Healthcare, Amersham, UK) and the plates were incubated overnight at 37°C in a humidified atmosphere containing 5% CO_2_. The serum-containing medium was removed and replaced by 200 µL serum-free McCoy's 5a medium supplemented with 0.5% (w/v) BSA (Gibco, Grand Island, NY, USA), 2 mM L-glutamine and antibiotics (penicillin 100 units, streptomycin 100 units, amphotericin B 0.25 µg/mL final, Gibco, Grand Island, NY, USA). The plates were incubated for 4 hours at 37°C in a humidified atmosphere containing 5% CO_2_. 150 µL of the medium were removed and substituted by 150 µL of serum-free medium containing the different insulins or IGF-1 (Cell Sciences, Canton, MA, USA) at the indicated concentrations and the plates were incubated at 37°C in a humidified atmosphere containing 5% CO_2_. After 19 hours 10 µL of [2-^14^C]-thymidine solution (>50 mmol/L, 3.7 MBq/mL) diluted in serum-free McCoy's 5a medium were added per well to yield a final concentration of 500 nCi/mL and the plates were incubated for 6 hours at 37°C in a humidified atmosphere containing 5% CO_2_. ^14^C-thymidine incorporation was measured in a Wallac 1450 Micro Beta Trilux Scintillation counter. Dose-response curves were obtained by testing ten different concentrations of the ligands with every concentration tested by octuplicate samples.

### Statistic Analysis

For each experiment, IC_50_ or EC_50_ values were obtained using the 4-parameter logistic model according to Ratkowsky and Reedy (1986). The adjustment was obtained by non-linear regression using the Levenberg-Marquardt algorithm in SAS v9.1.3 software via Biost@t-Speed V2.0-LTS internal application. If necessary, lower and upper asymptotes were set to 0 and 100, respectively.

Statistical analysis was performed with one-way analysis of variance (ANOVA), followed by a Bonferroni comparison or data were analyzed with a paired t-test using GraphPad Prism 5.02 (San Diego, CA, USA).

## Results and Discussion

### Metabolic Signaling and Activity of Insulin Glargine and Its Metabolites IM, M1, and M2

Previous studies have shown that insulin analogs differ only in potency but produce identical maximal responses. Accordingly, a good correlation between the potency in insulin receptor binding, induction of autophosphorylation and stimulation of lipid synthesis has been observed for insulin analogs [34]. Relative to human insulin, insulin glargine and its metabolite M1 demonstrated a reduced insulin receptor binding of 86% and 78%, respectively, and decreased lipid synthesis of 60% and 88%, respectively [12].

**Figure 2:**
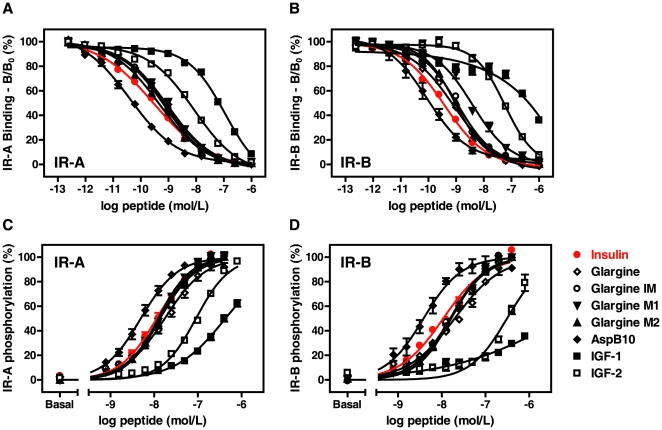
Binding and signaling of insulin glargine and its metabolites to the human insulin receptor isoform A and B. Binding of the insulin analogs to the human IR-A (**A**) or IR-B (**B**) was analyzed in a competitive binding assay using SPA technology. The binding of a constant concentration of [^125^I]insulin to plasma membranes from CHO cells overexpressing either IR-A or IR-B was measured in presence of increasing concentrations of unlabeled competing ligand after incubation at room temperature for 12 h. All data has been corrected for non-specific binding and are expressed as percentage of [^125^I]insulin in absence of competing ligand. To analyze the insulin-stimulated activation and subsequent autophosphorylation of the insulin receptor CHO cells overexpressing the human IR-A (**C**) or IR-B (**D**) were stimulated for 15 min at 37°C with increasing concentrations of peptides, then the cells were fixed with 3.7% PFA and the amount of phosphotyrosines was analyzed via In-Cell Western. The data represent mean values ± SEM of at least 3 individual experiments measured in quadruplicate.

First, we analyzed the binding characteristics of insulin glargine and its metabolites M1, M2, and IM to IR-A and IR-B. Using plasma membranes from CHO cells that overexpress the receptor isoforms, the IC_50_ value for competition of unlabeled ligand with a constant concentration of radioactively labeled human insulin were determined as a measure of affinity. The results are shown in Table 1 with representative competition curves given in Figure 2 A and B. Both IR isoforms bound human insulin with high affinity that did not significantly (p = 0.23) differ between IR-A (IC_50_ = 0.49 nmol/L) and IR-B (IC_50_ = 0.57 nmol/L). In contrast, IR-A displayed a considerably higher affinity for IGF-1 (2.7-fold) and IGF-2 (7.5-fold) than IR-B, but the affinity of IR-A and IR-B for IGF-1 were approximately 130- and 300-fold, respectively, less potent than those for human insulin. Both isoforms bound IGF-2 with higher affinity than IGF-1 but with lower affinity than human insulin. These data are consistent with results published previously [35]–[39] and demonstrated the validity of our experimental system.

**Table 1:** Summarized *in vitro* data for insulin and glargine metabolites.

Analog	IR-A affinity	IR-B affinity	IR-A auto-phosphorylation	IR-B auto-phosphorylation	Metabolic potency	IGF1R affinity	IGF1R auto-phosphorylation	Mitogenic potency
	IC_50_	IC_50_	EC_50_	EC_50_	EC_50_	IC_50_	EC_50_	EC_50_
	(nmol/L)	(nmol/L)	(nmol/L)	(nmol/L)	(nmol/L)	(nmol/L)	(nmol/L)	(nmol/L)
**Human insulin**	0.49±0.04	0.57±0.02	11.0±1.3	11.7±1.4	0.045±0.003	289±53.3	447±38.7	12.25±0.27
**Glargine**	0.83±0.08	1.10±0.12	18.4±2.4	23.6±2.1	0.066±0.005	63.2±19.9	87.5±10	1.61±0.26
**Glargine IM**	0.78±0.03	1.08±0.06	23.1±2.7	20.9±1.3	0.098±0.012	80.0±10.2	179±19.6	3.75±0.31
**Glargine M1**	1.02±0.03	1.35±0.20	18.6±2.5	18.1±1.7	0.139±0.009	649±31.9	644±56.9	16.25±2.35
**Glargine M2**	0.93±0.16	1.09±0.06	19.2±1.6	21.4±2.3	0.087±0.007	427±20.6	485±43.6	17.90±6.50
**[Asp^B10^]insulin**	0.06±0.01	0.21±0.03	5.1±0.7	3.32±0.78	0.031±0.005	104±12.8	72.7±7.6	1.52±0.15
**IGF-1**	64.5±5.1	171±50	449±61.7	>1,000	19.01±0.93	0.89±0.19	2.9±0.4	0.22±0.05
**IGF-2**	6.2±0.34	46.6±7.8	85.4±5.5	384±68.4	-	6.68±2.24	-	-

Data are means ± SEM. All analogs were tested at least three times on different days. Activity was determined within each experiment and then averaged to yield a single reported mean value. IM – intermediate ([Gly^A21^,Arg^B31^]insulin), M1 – metabolite 1 ([Gly^A21^]insulin), M2 – metabolite 2 ([Gly^A21^,des-Thr^B30^]insulin).

Insulin glargine and its metabolites IM, M1, and M2 showed no difference in their respective binding affinities to the two IR isoforms and were only 40–50% less active than human insulin. In contrast, [Asp^B10^]insulin showed significant isoform selectivity, being 3.5-fold more active towards IR-A (p<0.01), and was 8.2- and 2.7-fold more potent in binding to IR-A and IR-B, respectively, relative to human insulin.

Insulin receptor autophosphorylation was studied using CHO cells overexpressing either IR-A or IR-B. For detection an immunocytochemical microplate assay (In-Cell Western) based on two-colour fluorescence was applied [31]. The observed EC_50_ values for stimulation of autophosphorylation of the IR isoforms with human insulin, insulin glargine, its metabolites IM, M1 and M2 and IGF-1 and IGF-2 correlated well with their binding affinities to the corresponding receptors (Tab. 1, Fig. 2 C and D). For human insulin, insulin glargine and its metabolites the EC_50_ values were comparable for IR-A and IR-B with insulin glargine and its metabolites exhibiting a 30–50% decrease in activity towards both isoforms relative to human insulin. IGF-1 and more potently IGF-2 were capable in stimulating the autophosphorylation of IR-A. In contrast, stimulation of IR-B autophosphorylation was less pronounced with IGF-1 (EC_50_ value >400 nmol/L) and IGF-2 (EC_50_ value = 384±68 nmol/L). The most potent stimulant for both IR-A and IR-B autophosphorylation was [Asp^B10^]insulin with comparable EC_50_ values, which did not reflect the differences observed in the IR isoform affinities [12].

For determination of the metabolic activity primary rat adipocytes were used that possess a high insulin sensitivity and responsiveness. They reflect the physiological situation of insulin target cells with regard to signal transduction processes and regulation of glucose and lipid metabolism (e.g. glucose uptake and esterification into lipids) more closely than cultured muscle cells and adipocyte cell lines. mRNA expression of IR-B is significantly higher than that of IR-A in rat epididymal white adipose tissue from which primary adipocytes are derived [23]. By using fully differentiated and maximally insulin-sensitive adipocytes from young rats, any difference in metabolic activity between human insulin, insulin glargine and glargine metabolites is due to differential interaction with the IR-B rather than the IR-A.

Assaying lipid synthesis with primary rat adipocytes for the measurement of metabolic activity is based on the incorporation of radiolabelled glucose into total lipids. This pathway is under the strict control of insulin at the low glucose concentrations used.

**Figure 3:**
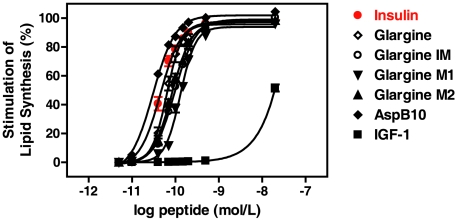
Metabolic activity of insulin glargine and its metabolites in rat adipocytes. To compare the metabolic activity of glargine and its metabolites insulin-stimulated lipid synthesis in isolated primary rat adipocytes was analyzed by incorporation of [3-^3^H]glucose into toluene-extractable lipids and subsequent measurement of radioactivity by liquid scintillation counting. The adipocytes were treated with increasing concentrations of insulin, analogs, metabolites or IGF-1 for 90 min at 37°C in the presence of [3-^3^H]glucose. Each point represents the mean ± SEM of five different adipocyte preparations with activity measurements done in duplicate.

As expected on the basis of previously reported data on the stimulation of lipid synthesis in rat adipocytes [12], [Asp^B10^]insulin and IGF-1 exerted the highest and lowest metabolic activities, respectively, among all proteins tested, with EC_50_ value of 0.2-fold lower and 422-fold higher, respectively, relative to insulin. Furthermore, insulin glargine and M1 were reported to exhibit 60% and 88%, respectively, of the metabolic activity of human insulin [12]. These findings were confirmed in the present study as reflected in the considerable right-ward shifts of the concentration-response curves for insulin glargine and M1 *vs*. human insulin resulting in 1.4- and 3.0-fold higher EC_50_ values, respectively (Tab. 1, Fig. 3). The glargine metabolites M2 and IM, here investigated for biological activity for the first time, were also less active (1.2- and 1.4-fold) than insulin glargine but more active than M1 (0.6-fold lower EC_50_ values). The maximal responses in stimulating lipid synthesis are the same for human insulin, insulin glargine and its metabolites M1, M2 and IM (15- to 20-fold above basal, depending on the batch of adipocytes used).

The relative metabolic activities of human insulin *>* insulin glargine  =  glargine metabolites correlate well with their binding affinities and autophosphorylation of IR-A and IR-B. This is compatible with previous studies demonstrating that ligand affinity and activation (i.e. autophosphorylation) of the IR are the key events for initiation of downstream signaling to the metabolic effector systems of insulin and its analogs [6], [40].

The maintenance of binding, autophosphorylation and metabolic activities of insulin analogs with modified carboxy-terminus confirm the previous findings that amino acid modifications at the insulin B-chain beyond position B25, in particular the B26-B30 region, do not play a critical role in its recognition by both isoforms of the IR [41], [42] and thus in metabolic signaling in insulin target cells [6], [43]. The slightly lower binding, autophosphorylation and metabolic activities of M1 relative to those of human insulin are in agreement with the previous suggestion that the minor structural change caused by loss of the salt bridge between Asn^A21^ and Arg^B22^ leads to a (small) reduction in IR affinity [44]. In consequence, the slight reduction in metabolic activity of insulin glargine *vs.* human insulin derives predominantly from altered structural features at the carboxy-terminus of the B-chain and rather than from the amino acid substitution at A21.

Taken together, the present data clearly demonstrate that the major metabolites of insulin glargine detected in rats, dogs, and humans [9], [10] are metabolically active. Since a ratio of 50∶50 for parent compound and M1/M2 was reported in tissue samples derived from the site of injection in humans [10] and *in vitro* metabolism of insulin glargine resulted into 46% to 98% appearance of M1 [11], it is reasonable to assume that the metabolites of insulin glargine will contribute to the blood glucose lowering profile observed after administration of insulin glargine, dependent on the extent of their generation at the subcutaneous injection site and in plasma in the course of carboxypeptidase action [11].

### Mitogenic Signaling and Activity of Insulin Glargine and Its Metabolites IM, M1, and M2

Although the predominant physiological function of insulin is the regulation of glucose and lipid metabolism *via* the insulin signaling cascade, the hormone is able to stimulate proliferation in different tissues and primary cell lines as well as in tumors and tumor-derived cell lines through either IR or IGF1R signaling pathways [34].

**Figure 4:**
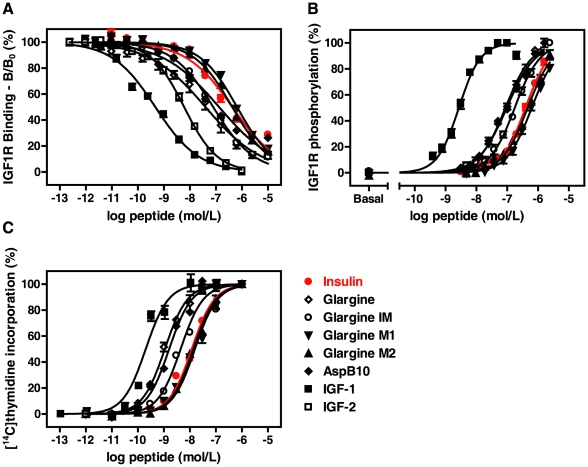
Activity of insulin analogs on the human IGF1R and mitogenic potential in Saos-2 cells. Analysis of binding to the human IGF1R using [^125^I]IGF-1 as radioactive ligand (**A**) and analysis of the insulin-stimulated autophosphorylation of the human IGF1R overexpressed in MEF cells (**B**) were done as described in figure 2 and Materials and Methods. Data are given as means ± SEM. (**C**) To compare the mitogenic potential of the insulin analogs with that of human insulin, DNA synthesis was determined by [^14^C]thymidine incorporation in Saos-2 cells cultured in Cytostar-T scintillation microplates. Confluent cells were starved for 4 h and then incubated for 19 h with increasing concentrations of IGF-1, insulin or analog in serum free medium. [^14^C]thymidine was added for further 6 h and the radioactivity measured in a Wallac Micro Scintillation counter. Data are given as means ± SEM from octuplicate sample.

Insulin glargine resembles [Asp^B10^]insulin in increasing the incorporation of radiolabelled thymidine into DNA in Saos-2 cells with comparable EC_50_ values, which are 7- to 10-fold lower than those for human insulin (Tab. 1, Fig. 4 C). In addition, the affinities for IGF1R and the EC_50_ values for IGF1R autophosphorylation of insulin glargine and [Asp^B10^]insulin were comparable and moderately greater, respectively (Tab. 1, Fig. 4 A and B). Relative to human insulin, insulin glargine and [Asp^B10^]insulin were about 5-fold more potent in the stimulation of IGF1R autophosphorylation. Relative to IGF-1, human insulin, insulin glargine and [Asp^B10^]insulin were about 150, 30 and 25 times less potent. These findings are in agreement with the observations described by Kurtzhals *et al.* [12] and Seipke *et al.* [45]; the latter have been intensively evaluated by European Medical Evaluation Agency during the approval of insulin glargine for marketing authorization [46]. This may be explained by involvement of the carboxy-terminal part of the insulin B-chain in interaction with the IGF1R, as amino acid substitutions at positions B28-B32, in particular, the number and position of basic residues, have been shown to affect the binding affinity to the IGF1R [47]. Consequently the two major metabolites M1 and M2 were significantly less mitogenic than glargine and comparable to normal insulin. The sequential removal of the amino acids from the carboxy-terminus of the B-chain led to a decreased affinity towards IGF1R and thus resulted in higher EC_50_ values for IGF1R autophosphorylation (Tab. 1, Fig. 4 A and B). The intermediate IM, in which only Arg^B32^ is missing, had a 2-fold higher potency relative to human insulin. However, for M1 and M2, the IGF1R affinity was significantly reduced and EC_50_ values for IGF1R autophosphorylation were >400 nmol/L. The mitogenic potencies of IM, M1 and M2 were reduced relative to insulin glargine, with an activity of M1 and M2 as low as human insulin (Tab. 1, Fig. 4 C).

A correlation between IGF1R autophosphorylation and cell proliferation has been recently assumed by Shukla *et al.* [15], who observed an enhanced growth of the malignant cell line MCF7 after insulin glargine stimulation. The authors have observed 10% and 50% increases of cellular proliferation at 0.31 and about 15 nmol/L. This effect seemed to be linked to activation of the IGF1R and the MAPK pathway, since siRNA knockdown of IGF1R decreased the response [15]. Unfortunately, no dose-response curve of IGF1R autophosphorylation as well as no proliferation data from siRNA knockdown of IR have been presented so far. Other breast cancer cell lines containing IR and IGF1R did not respond to human insulin (BT474, T47D, ZR75-1, MDA-MB231, HCC1937). Furthermore, with the benign cell line MCF10 human insulin and insulin glargine did not show any difference [15]. Several studies have been dedicated to the growth response of MCF7 and other tumor cell lines towards insulin and insulin analogs. Staiger *et al.* [14] found no evidence for an increased mitogenic activity of insulin glargine relative to human insulin. Liefvendahl *et al.* [48] tested insulin glargine on MCF-7 and SKBR-3 cells and found a slightly increased mitogenic activity of insulin glargine without statistical significance. Weinstein *et al.* [49] tested the potency of insulin, insulin glargine and the two insulin analogs insulin detemir and insulin lispro in MCF-7 cells, HCT-116 (colorectal cancer) and PC-3 (prostate cancer) cells and showed an increased mitogenic potency of insulin glargine and insulin detemir.

These data are likely to support an involvement of the IGF1R signaling cascade but are not yet conclusive. Consequently it is hard to draw any conclusion from these *in vitro* results for the role of an insulin-mediated IGF1R-promoted cell growth *in vivo*.

Linking mere IGF1R activation by insulin glargine and by [Asp^B10^]insulin *in vitro* and tumor-promoting activity *in vivo* ignores the fact that the IR activity profile of both molecules differ completely. Whereas [Asp^B10^]insulin has a high affinity for both receptor isoforms IR-A and IR-B resulting in a decreased EC_50_ value for receptor autophosphorylation and in a prolonged occupancy leading to increased mitogenicity [50], insulin glargine is less active on both IR-A and IR-B relative to human insulin and has a dissociation rate similar to that of human insulin [12], [51]. Taken together, this raises the possibility that the higher tumor incidence found in rats in the course of [Asp^B10^]insulin treatment is predominantly mediated by the IR signaling axis involving both IR-A and IR-B (Tab. 1). Most important the data presented indicate that for insulin glargine and its metabolites mitogenic activity higher than human insulin can be excluded for the IR-mediated pathway.

Importantly, healthy subjects and patients with type 1 diabetes who received insulin glargine injections showed free serum insulin glargine levels between 70 to 90 pmol/L with peak levels of about <200 pmol/L after injection [52]–[55]. The therapeutic insulin glargine concentrations reported so far are 80- to 200-fold below the total plasma concentrations of IGF-1. Notably, up to 99% of the circulating IGF-1 is bound to the IGF-binding proteins 1–6 in human plasma [13], [56]. Therefore, the concentration of free IGF-1 may be within the range of the plasma concentration of insulin glargine. However, despite the putative comparable plasma concentrations the ∼100-fold reduced affinity of insulin glargine towards IGF1R compared to that of IGF-1 makes a competition even of non-metabolized insulin glargine with endogenous IGF-1 at the receptor unlikely. Furthermore, the concentration of free IGF-1 may be elevated during certain diseases and metabolic states, which are known to considerably reduce the expression of IGF-binding proteins [57], [58] making interaction of insulin glargine with the IGF1R in course of displacement of IGF-1 even less likely.

Insulin glargine has been evaluated extensively in a 2-year life-time carcinogenicity study in rats without evidence of tumor-promoting activity greater than human insulin [59]. In these studies plasma insulin concentrations were 15 to 25 nmol/L, covering parent compound and active metabolites. In consequence, actual glargine concentrations may be overestimated, the extent of which depends on the degree of proteolytic degradation at both the injection site and the serum. Quantitative assessment of the insulin glargine metabolism in humans is evolving, and will bring further clarification.

### Conclusion

The present data unequivocally demonstrate similar metabolic signaling *in vitro* and activity in rat adipocytes of insulin glargine and its major metabolites with comparable engagement of IR-A and IR-B. In contrast, the insulin glargine metabolites were found to be less potent in mitogenic signaling and less active in Saos-2 proliferation assay compared to insulin glargine. The formation of insulin glargine metabolites may help to explain the established efficacy and safety of insulin glargine as observed in clinical practice and particularly in long-term clinical trials.
